# The Malone antegrade continence enema for treating adult constipation and fecal incontinence: a systematic review of the literature

**DOI:** 10.1007/s00384-025-05022-5

**Published:** 2026-01-03

**Authors:** Gaetano Gallo, Veronica De Simone, Alex Bruno Bellocchia, Salvatore Sorrenti, Alberto Realis Luc, Giuseppe Clerico, Roberto Sorge, Pierpaolo Sileri, Mario Trompetto, Gianpiero Gravante

**Affiliations:** 1 Faculty of Medicine and Surgery, Colorectal Surgery Unit, IRCCS San Raffaele Scientific Institute, Vita-Salute University, Via Olgettina 60, 20132 Milan, Italy; 2 Department of General Surgery, Maria Vittoria Hospital, Turin, Italy; 3 Department of Surgery, Sapienza” University of Rome, Rome, Italy; 4 Department of Colorectal Surgery, S. Rita Clinic, Vercelli, Italy; 5 Department of Human Physiology, Laboratory of Biometry, University of Tor Vergata in Rome, Rome, Italy; 6 Department of General Surgery, Azienda Sanitaria Locale ASL Lecce, Casarano, Italy

**Keywords:** Antegrade continence enema, Fecal incontinence, Constipation, Neurogenic bowel dysfunction, Colostomy

## Abstract

**Purpose:**

The Malone antegrade continence enema (MACE) offers a minimally invasive and potentially reversible option for managing chronic constipation and fecal incontinence (FI). This systematic review evaluates its efficacy, safety, and long-term outcomes in adults.

**Methods:**

A comprehensive search was conducted across PubMed, EMBASE, and CENTRAL databases up to April 2025 to identify studies on MACE in adults. Study quality was assessed using the Newcastle–Ottawa scale. The primary outcome was the proportion of patients continuing MACE at follow-up (treatment success); failure was defined as conversion to definitive colostomy.

**Results:**

Seventeen studies with 404 patients were included. Study quality was rated moderate to good. The most common indications were neurological disorders (25.8%), prior surgeries (16.8%), idiopathic constipation (14.2%), and traumatic spinal injuries (11.6%). Techniques included terminal ileal loop (37.9%), percutaneous endoscopic cecostomy (26.0%), and appendicostomy (24.8%). Minor stoma-related complications were most frequent (39.1%), followed by fecal leakage (16.2%) and stoma stenosis (11.3%). Median follow-up was 28.5 months. At final follow-up, 75.1% of patients continued using MACE, while 9.8% required colostomy. Satisfactory outcomes were reported by 60%–83% of patients, with improvements in symptoms and quality of life.

**Conclusions:**

MACE is a safe and effective option for adults with refractory constipation or FI, especially in those aiming to avoid a permanent colostomy.

## Introduction

Physiological bowel movements require normal colonic transit, intact anorectal sensitivity, adequate expulsive forces, and coordinated pelvic floor function. When any of these mechanisms are impaired, patients may experience constipation or fecal incontinence (FI). Defecatory disorders affect up to one-quarter of the general adult population [1–3], and when present, they can significantly impair quality of life (QOL) [4]. Conservative treatments include dietary modifications, oral laxatives, bulking agents, suppositories, and biofeedback therapy [5]. When these measures fail, surgical interventions may be considered. These include transanal colonic irrigation (TAI), percutaneous tibial nerve stimulation, and sacral neuromodulation [5, 6]. Colonic resections—either total or segmental—and stoma formation represent last-resort options [7], although their outcomes are variable and often associated with considerable morbidity [8, 9]. Among surgical options, the Malone antegrade continence enema (MACE) offers a distinctive approach by enabling antegrade colonic irrigation, which may provide more predictable and complete bowel emptying and improve patient autonomy and quality of life, particularly in those who are refractory to conservative therapies.

MACE was introduced as a less invasive and potentially reversible surgical alternative for managing chronic constipation and FI [10]. The principle involves performing antegrade enemas through a conduit into the proximal colon, facilitating mechanical irrigation and stimulating colonic propagating contractions. This dual mechanism enables complete and regular evacuation, helping prevent both constipation and FI [11]. Initially developed for pediatric patients, MACE has since been adapted for use in adults with similar bowel dysfunctions [12]. The technique has evolved over time to include minimally invasive laparoscopic and endoscopic approaches [13, 14]. Reported outcomes have been encouraging: between 47 and 100% of patients continue irrigation in the medium and long term, while failure rates, defined as conversion to permanent colostomy, range from 0% to 24% [10]. In recent years, evolving techniques have included minimally invasive laparoscopic and endoscopic approaches. Such developments aim to optimize stoma creation, reduce complications, and expand indications, reflecting a growing interest in tailoring MACE to adult populations and improving long-term functional outcomes.

The aim of this study is to perform a systematic review of MACE procedures in adult patients. We present pooled success and failure rates and provide a detailed analysis of complications associated with different MACE techniques.

## Materials and methods

A review protocol was registered in advance on the International Prospective Register of Systematic Reviews (PROSPERO; registration number: CRD420251025965; https://www.crd.york.ac.uk/PROSPERO/view/CRD420251025965). The work has been reported in line with PRISMA (Preferred Reporting Items for Systematic Reviews and Meta-Analyses) and TITAN (Transparency in the Reporting of Artificial Intelligence) guidelines [15, 16].

Inclusion criteria comprised all published studies where MACE was performed in the adult population (older than 16 years) for the treatment of constipation and/or FI. Exclusion criteria included pediatric studies, studies where MACE was performed during childhood and adolescence and that report outcomes in adulthood [17, 18], and studies focusing on outcomes other than constipation or FI. All study types were considered eligible—including retrospective, prospective, observational studies, and clinical trials—with the exception of case reports. Given the expected heterogeneity across study designs and outcome measures, both retrospective and prospective studies were included. Only articles published in English, or those for which a reliable English translation was available, were included.

The literature search was conducted using the biomedical databases PubMed, Embase, and the Cochrane Central Register of Controlled Trials (CENTRAL) from inception to August 2025. The PubMed search strategy combined the following terms: (“antegrade” AND (“continent” OR “continence”) AND “enema”) OR (“antegrade continence enema”), applying filters for “Humans” and “All Adult (16 + years).” The Embase search was performed using an equivalent strategy adapted to Emtree terminology. The CENTRAL database was queried with the term “antegrade continence enema” across titles, abstracts, and full texts. The Current Controlled Trials database (www.controlled-trials.com) was also screened to identify ongoing randomized trials. Additionally, the reference lists of relevant articles and prior reviews were screened for further eligible studies.

Titles and abstracts were screened for relevance, and full texts of potentially eligible studies were independently reviewed by two authors (GG and GGr) with any disagreements resolved by discussion or consultation with a third author (ABB). Data from eligible studies were extracted and compiled into a centralized database. The methodological quality of the included studies was evaluated using the Newcastle–Ottawa scale (NOS) for cohort studies [19]. The NOS assigns up to nine points across three domains: selection (four points: representativeness of the exposed cohort, selection of the non-exposed cohort, ascertainment of exposure, and demonstration that the outcome was not present at baseline), comparability (two points: adjustment for the most important confounder and for additional confounders), and outcome (three points: adequacy of outcome assessment, length of follow-up sufficient for outcomes to occur, and adequacy of follow-up of cohorts). Each item was judged as either fulfilled (awarding a star) or not fulfilled. Two authors (GGa and GGr) independently assessed all studies, with disagreements resolved by discussion with the third author (ABB). Based on the total score, studies were categorized as high quality (≥ 7 points), moderate quality (5–6 points), or low quality (< 5 points).

The primary outcome of this systematic review was to assess the long-term clinical efficacy of MACE in adult patients, defined in a patient-centered manner as both the sustained use of antegrade colonic irrigation and meaningful improvements in bowel function and QOL. Traditional measures, such as continued MACE use and the need for definitive colostomy, were included as objectively measurable endpoints; however, these may not fully capture the functional and experiential benefits from the patient perspective. Functional outcomes were extracted as reported, acknowledging the use of diverse scoring systems and QOL instruments, which limited the possibility of direct comparison. Secondary outcomes included the safety of the procedure (type and frequency of postoperative complications) and functional outcomes assessed using validated symptom scores and QOL instruments. Where sufficient data were available, descriptive pooled estimates were calculated, while studies with missing or unclear data were excluded from quantitative synthesis. Because all included studies were single-arm series without comparators, and given the heterogeneity expected (follow-up, indications, technique), a formal meta-analysis and associated bias assessments (e.g., funnel plots) were not applicable.

## Results

**Fig. 1:**
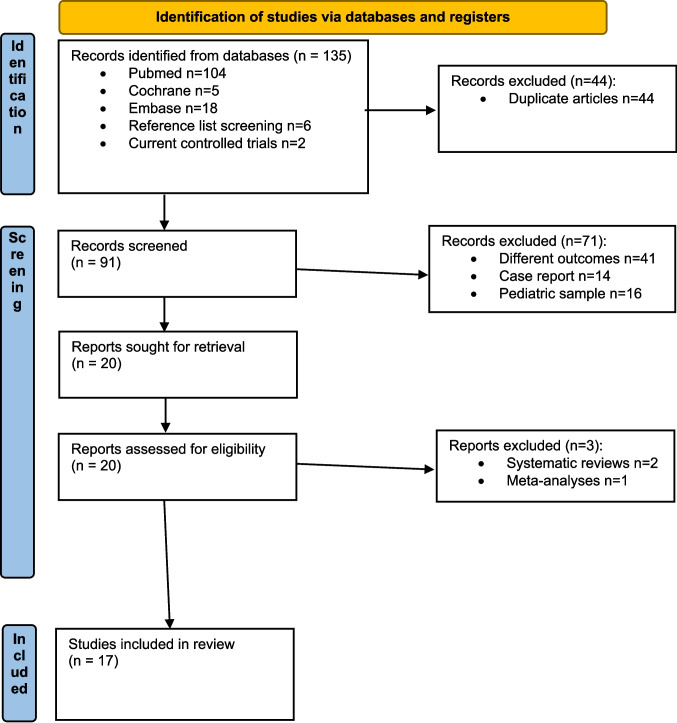
Flow-chart showing the retrieval, analysis, and selection process of articles included in the systematic review

Seventeen articles were included in the final analysis (Fig. 1) [12–14, 20–33]. Eleven were retrospective studies [12, 14, 20, 26, 32, 33], and six were prospective (Table 1) [13, 27–31]. All but two studies were conducted in European countries [23, 33], and all but one were single-center series (Table 1) [20]. According to the NOS, 11 studies were rated as “moderate-to-good” quality (6/9 points), four as moderate (5/9 points), and two as good quality (7/9 points) (Table 2). Most studies used validated outcome measures, had sufficient follow-up duration to assess long-term results, and reported complete follow-up data for all patients (Table 2). Common limitations included the absence of control groups, small sample sizes, and the resulting inability to perform subgroup analyses (e.g., comparisons between indications—FI vs. constipation—or between surgical techniques) (Table 2).

**Table 1:** Overview of study characteristics. *FI*, fecal incontinence; *APP*, appendicostomy; *IlAPP*, ileal neoappendicostomy; *CeA*, cecostomy; *PEC*, percutaneous endoscopic caecostomy. *Lees et al. reported one patient with a left colonic conduit due to a previous right hemicolectomy; this patient is not included in the table

Study	Year	Country	Setting	Type of study	No. of patients	Sex (men–women)	Age (years)	Indication to surgery	Technique used
Hill et al. [12]	1994	UK	Single-center	Retrospective	6	-	23–47	Constipation alone	6 APP
Krogh et al. [21]	1998	Denmark	Single-center	Retrospective	16	6:10	41 (20–68)	Constipation alone, FI alone	12 APP, 4 IlAPP
Rongen et al. [31]	2001	Netherlands	Single-center	Prospective	12	4:8	43 (17–66)	Constipation alone	7 APP, 5 IlAPP
Teichman et al. [33]	2003	USA	Single-center	Retrospective	6	5:1	32 (19–47)	Constipation alone or with FI	6 APP
Lees et al. [32]	2004	UK	Single-center	Retrospective	31*	9:26	35 (16–72)	Constipation alone	20 APP, 7 IlAPP, 4 CeA*
Hirst et al. [22]	2005	UK	Single-center	Retrospective	20	0:20	44 (20–65)	Constipation alone or with FI	13 APP, 7 CeA
Portier et al. [28]	2006	France	Single-center	Prospective	28	-	55 ± 13	Constipation with FI	7 APP, 15 IlAPP, 6 CeA
Lefèvre et al. [29]	2006	France	Single-center	Prospective	25	8:17	47 (16–76)	FI alone	7 App, 18 IlAPP
Uno [23]	2006	Japan	Single-center	Retrospective	15	15:5	67 ± 19	Constipation alone	15 PEC
Altomare. et al. [27]	2007	Italy	Single-center	Prospective	11	4:7	48 (24–70)	Constipation with FI	11 Marsh and Kiff
Meurette et al. [30]	2010	France	Single-center	Prospective	25	3:22	51 ± 11	Constipation alone	3 APP, 4 IlAPP, 18 CeA
Chéreau et al. [24]	2011	France	Single-center	Retrospective	75	21:54	48 (18–81)	FI alone	7 APP, 68 IlAPP
Duchalais E. et al. [13]	2015	France	Single-center	Prospective	21	4:21	47 (20–71)	Constipation alone, FI alone	21 PEC
Sturkenboom R. et al. [14]	2018	Netherlands	Single-center	Retrospective	18	3:15	39 ± 18 FC 48 ± 20 FI	Constipation alone, FI alone	12 APP, 6 IlAPP
Ricard J et al. [20]	2019	France	Multicentric	Retrospective	69	-	49 (19–75)	Constipation alone, FI alone	69 PEC
Brinas P. et al. [25]	2020	France	Single-center	Retrospective	23	10:13	42 (28–70)	Constipation alone, FI alone	23 IlAPP
Spinelli M. et al. [26]	2021	Italy	Single-center	Retrospective	3	3:0	49 ± 12	Not specified	3 IlAPP

**Table 2:**
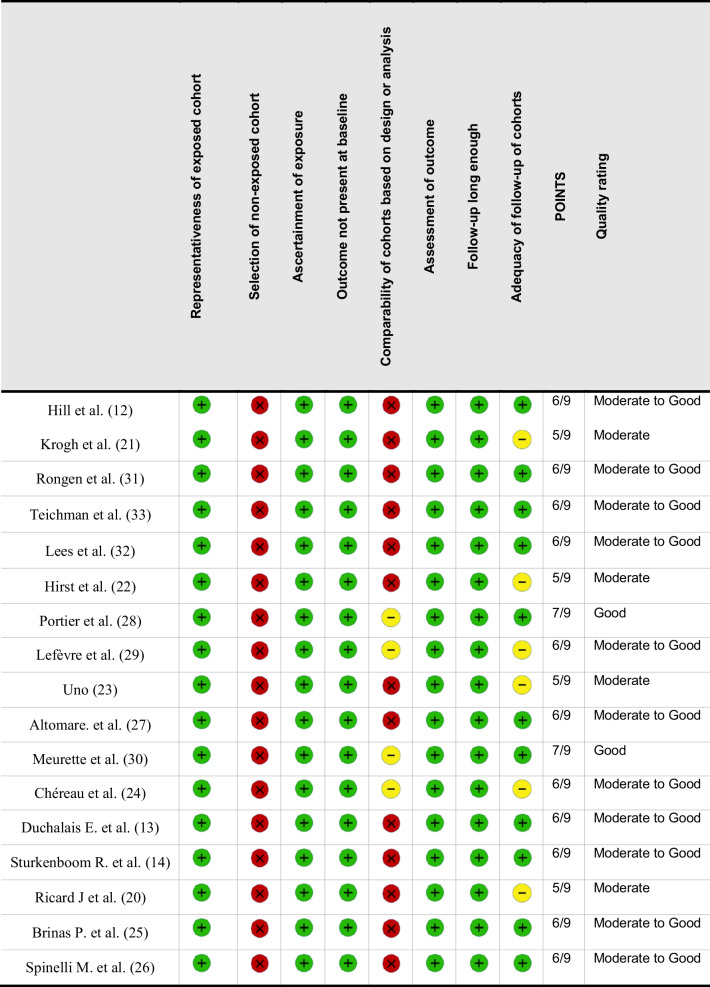
Newcastle–Ottawa scale of included studies

### Indications for MACE

A total of 404 adult patients underwent MACE procedures, with individual series ranging from 3 to 75 patients (Table 1). Patients were predominantly female (219/301, 72.8%), except in three studies that showed a male predominance [23, 26, 33]. Three studies did not report patient gender (Table 1) [12, 20, 28].

The most frequent indication for MACE was constipation (183/371, 49.3%; 44.2%–54.4% 95% CI), followed by FI (143/371, 39.6%; 34.6%–44.6% 95% CI) (Table 3). Two studies did not clearly report indications and were therefore excluded from the pooled analysis [26, 28]. The most common underlying causes were neurological disorders (89/345, 25.8%; 21.2%–30.4% 95% CI), followed by previous surgeries (58/345, 16.8%; 12.9%–20.7% 95% CI), idiopathic etiologies (49/345, 14.2%; 10.5%–17.9% 95% CI), and traumatic spinal injuries (40/345, 11.6%; 8.2%–15.0% 95% CI) (Table 3). Two studies did not clearly report the causes of constipation or FI (*n* = 28) [28], or failed to specify them for all patients (*n* = 32) [32], and were likewise excluded from the pooled analysis.

**Table 3:** Pooled analysis of MACE indications, causes, and techniques used. Lees et al. reported one patient with a left colonic conduit due to a previous right hemicolectomy; this patient is not included in the table

	Number	Percentage	95% CI	References
Indications				
• Constipation	183/371	49.3%	(44.2%–54.4%)	[12, 13, 20–23, 28, 30–33]
• FI	147/371	39.6%	(34.6%–44.6%)	[20, 21, 24, 28, 29]
• FI with constipation	41/371	11.1%	(7.9%–14.3%)	[13, 20, 22, 27, 33]
Causes				
• Neurological disorders	89/345	25.8%	(21.2%–30.4%)	[13, 14, 20, 21, 23–27, 29, 30, 33]
• Previous surgery	58/345	16.8%	(12.9%–20.7%)	[20, 21, 24, 27, 29, 30]
• Idiopathic	49/345	14.2%	(10.5%–17.9%)	[12–14, 21, 27, 30, 31]
• Traumatic spinal injuries	40/345	11.6%	(8.2%–15.0%)	[14, 21, 23, 25–27, 29, 33]
• Anorectal malformations	39/345	11.3%	(8.0%–14.6%)	[13, 20, 24, 27, 29]
• Obstructed defecation syndrome	24/345	7.0%	(4.3%–9.7%)	[14, 21, 22]
• Obstetric injuries	16/345	4.6%	(2.4%–6.8%)	[24, 29]
• Radiotherapy	8/345	2.3%	(0.7%–3.9%)	[20]
• Others	45/345	13.0%	(9.5%–16.5%)	[13, 14, 23, 24, 31]
Technique				
• Ileal neoappendicostomy	153/404	37.9%	(33.2%–42.6%)	[21, 24–26, 28–32]
• Percutaneous endoscopic cecostomy	105/404	26.0%	(21.7%–30.3%)	[13, 20, 23]
• Appendicostomy	100/404	24.8%	(20.6%–29.0%)	[14, 21, 22, 24, 27–33]
• Cecal flap	35/404	8.7%	(6.0%–11.4%)	[14, 28, 30]
• Modified Marsh and Kiff ileostomy	11/404	2.7%	(1.1%–4.3%)	[10, 27]

### Techniques and short-term outcomes

Five different surgical techniques were employed across the included studies, all of which reported the number of patients undergoing each technique (Table 3). The most commonly used method was the terminal ileal loop (ileal neoappendicostomy; 153/404, 37.9%; 33.2%–42.6% 95% CI), followed by percutaneous endoscopic cecostomy (105/404, 26.0%; 21.7%–30.3% 95% CI) and appendicostomy (100/404, 24.8%; 20.6%–29.0% 95% CI—Table 3). The stoma was most frequently created in the right iliac fossa, though occasionally positioned at the umbilicus for cosmetic reasons [32, 33].

**Fig. 2:**
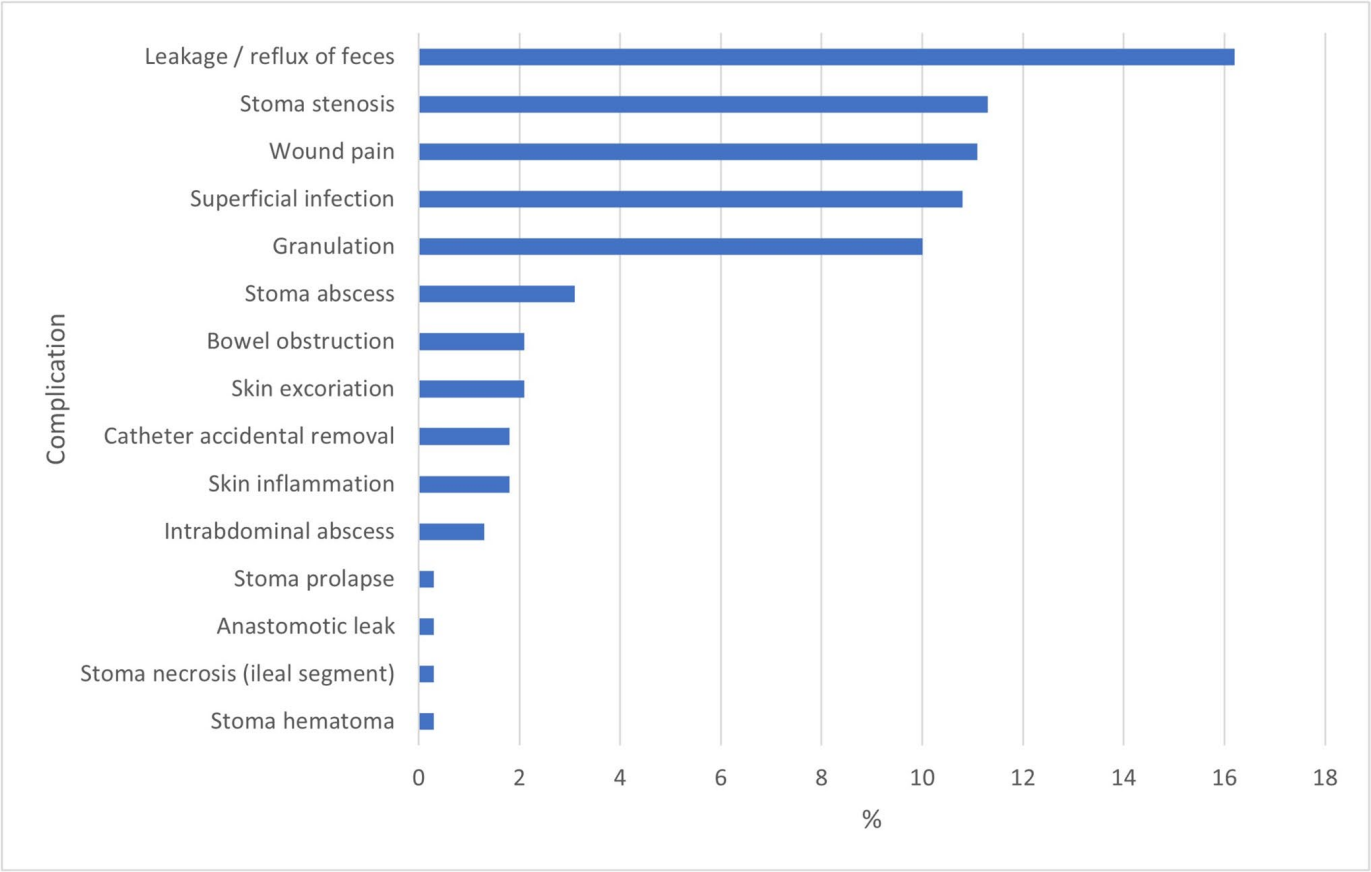
Bar chart showing complications’ pooled frequencies (%) in decrescent order

All but one study (Uno et al. [23]) reported postoperative complications (Table 4). Clavien–Dindo grade I–II complications (not requiring invasive treatment) were reported in 56.3% (51.4%–61.2% 95% CI) of patients (219/389), while grade III complications occurred in 14.4% (10.9%–17.9% 95% CI) of them (56/389). No grade IV or V complications were reported. There was substantial heterogeneity in the types of complications reported (Table 4, Fig. 2), and only a limited number of studies described technique-specific complications when multiple approaches were used [12, 13, 20, 25, 28, 29]. Overall, minor stoma-related complications—including superficial infections, granulation tissue, local pain, abscesses, excoriation, inflammation, and hematomas—were the most frequent, affecting 39.1% (152/389; 34.3%–43.9% 95% CI) of patients. These were followed by fecal leakage (63/389; 16.2%; 12.5%–19.9% 95% CI) and stoma stenosis (44/389; 11.3%; 8.2%–14.4% 95% CI) (Table 4). Stoma stenosis was significantly more likely after appendicostomy than ileal neoappendicostomy (9/29, 31.0% vs. 3/60, 5.0%; odds ratio = 6.3, 1.6–25.0 95% CI; Fisher’s exact test *p* = 0.001). No statistically significant differences were found between appendicostomy and cecostomy (9/29, 31.0% vs. 3/24, 12.5%; OR = 2.5, 0.6–10.3 95% CI; Fisher’s exact test *p* = 0.11), nor between cecostomy and ileal neoappendicostomy (3/24, 12.5% vs. 3/60, 5.0%; OR = 2.5, 0.9–26.5 95% CI; Fisher’s exact test *p* = 0.228).

**Table 4:** Short-term outcomes: pooled analysis of complications. *IlAPP*, ileal neoappendicostomy; *CeA*, cecostomy; *APP*, appendicostomy; *PEC*, percutaneous endoscopic caecostomy. *Only studies explicitly reporting complications by technique were included in the detailed analysis of technique-specific complication rates [24, 30]

Complications	References	Overall (*n* = 389)	95% CI	IlAPP (*n* = 60)*	95% CI	PEC (*n* = 90)	95% CI	APP (*n* = 29)*	95% CI	CeA (*n* = 24)*	95% CI
Stoma minor complications		152 (39.1%)	(34.3%–43.9%)								
• Wound pain	[13, 20]	43 (11.1%)	(8.0%–14.2%)	-	-	43 (47.8%)	(37.5%–58.1%)	-	-	-	-
• Superficial inf.	[13, 14, 20–22, 24, 31–33]	42 (10.8%)	(7.7%–13.9%)	-	-	15 (16.7%)	(9.0%–24.4%)	2 (6.9%)	(0.0%–16.1%)	-	-
• Granulation	[13, 20, 22]	39 (10.0%)	(7.0%–13.0%)	-	-	38 (42.2%)	(32.0%–52.4%)	-	-	-	-
• Abscess	[14, 25, 28, 31, 32]	12 (3.1%)	(1.4%–4.8%)	1 (1.7%)	(0.0%–5.0%)	-	-	1 (3.4%)	(0.0%–10.0%)	2 (8.3%)	(6.8%–9.8%)
• Excoriation	[22]	8 (2.1%)	(0.7%–3.5%)	-	-	-	-	-	-	-	-
• Inflammation	[30]	7 (1.8%)	(0.5%–3.1%)	-	-	-	-	-	-	-	-
• Hematoma	[24]	1 (0.3%)	(0.0%–0.8%)	-	-	-	-	-	-	-	-
Leakage/reflux of feces	[13, 14, 22, 24, 28, 30–32]	63 (16.2%)	(12.5%–19.9%)	4 (6.7%)	(0.4%–13.0%)	7 (7.8%)	(2.3%–13.3%)	5 (17.2%)	(3.5%–30.9%)	4 (16.7%)	(1.8%–31.6%)
Stoma stenosis	[12, 14, 21, 22, 24, 28–30, 32, 33]	44 (11.3%)	(8.2%–14.4%)	3 (5%)	(0.0%–10.5%)	-	-	9 (31.0%)	(14.2%–47.8%)	3 (12.5%)	(0.0%–25.7%)
Bowel obstruction	[24, 33]	8 (2.1%)	(0.7%–3.5%)	-	-	-	-	3 (10.3%)	(0.0%–21.4%)	-	-
Catheter accidental removal	[13, 22]	7 (1.8%)	(00.5%–03.1%)	-	-	2 (2.2%)	(0.0%–5.2%)	-	-	-	-
Intra-abdominal abscess	[25, 28]	5 (1.3%)	(0.0%–2.4%)	-	-	-	-	2 (6.9%)	(0.0%–16.1%)	-	-
Stoma prolapse	[24, 30]	3 (0.8%)	(0.0%–1.7%)	-	-	-	-	-	-	-	-
Stoma necrosis (ileal segment)	[25]	1 (0.3%)	(0.0%–0.8%)	1 (1.7%)	(0.0%–5.0%)	-	-	-	-	-	-
Anastomotic leak	[25]	1 (0.3%)	(0.0%–0.8%)	1 (1.7%)	(0.0; 5.0%)	-	-	-	-	-	-

### Long-term success and failure

**Fig. 3:**
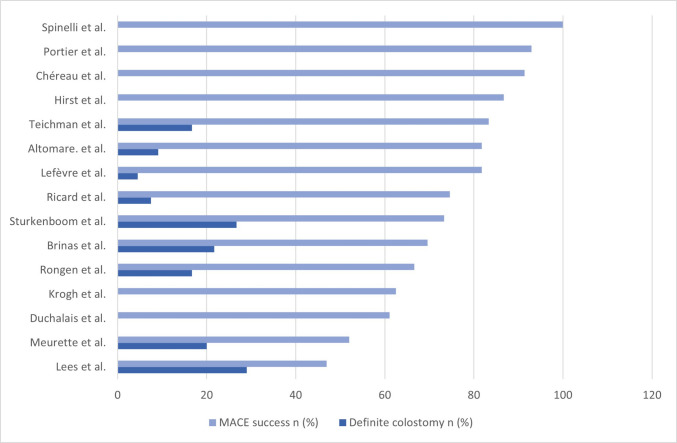
Bar chart showing success rates and failure rates (definite colostomy) for individual studies

The median follow-up was 28.5 months (range, 1–140 months) (Table 5, Fig. 3). A majority of patients (283/377; 75.1%, 70.7%–79.5% 95% CI) continued to use colonic irrigations at the end of follow-up (range, 48.4%–100%; Table 5). MACE failure, defined as conversion to permanent colostomy, was reported in 9.8% (6.8%–12.8% 95% CI) of patients (37/377). Major causes of failure included stoma-related complications such as stenosis, necrosis, and abscesses [25]. Other causes included progression of the underlying disease [27], persistence of symptoms [29], abdominal pain, and reflux or leakage from the stoma [21].

### Functional results

Since 1998, functional outcomes have been assessed using various symptom-specific and quality-of-life instruments (Table 5) [21]. Four studies did not report functional outcomes or did not use validated assessment tools [12, 23, 28, 32]. Constipation severity was evaluated using the Knowles-Eccersley-Scott Symptom (KESS) score [34] in five studies [13, 20, 25, 30, 34], and the Cleveland Clinic Constipation Score (CCCS) [35] in three studies [14, 27, 31]. Fecal incontinence was assessed with the Cleveland Clinic Incontinence Score (CCIS) [6] in six studies [6, 20, 22, 24, 25, 35], and with the Vaizey Score [36] in one study [14]. Stomal continence was measured using the Malone continence scale [37] in a single study [14]. QOL was assessed using the Gastrointestinal Quality of Life Index (GIQLI) [38] in five studies [13, 20, 24, 27, 30], the Fecal Incontinence Quality of Life (FIQL) score [39] in one study [25], the Short Form (SF) 36 Health Survey [40] in four studies [14, 22, 24, 29], and the Nottingham Health Profile [41] in one study [31]. The Zung self-rating depression scale [42] and the State-Trait Anxiety Inventory [43] were used to assess anxiety and depression in one study [31].

Satisfactory outcomes, defined as improved function and QOL, were reported in 60%–83% of patients (Table 5). However, no definitive conclusion could be drawn regarding whether MACE provided superior results for constipation or FI. Some studies suggested better outcomes in constipated patients [13, 14], while others favored incontinent patients [24, 29, 30]. In the study by Brinas et al. [25], KESS and CCIS scores did not significantly change postoperatively, though this result may have been influenced by the low number of patients who completed follow-up questionnaires.

## Discussion

Constipation and FI are disorders that significantly impact QOL, affecting approximately 10% of the general adult population [1, 3]. These conditions may occur individually, although their coexistence is common and clinically variable [44]. Initial management typically involves conservative medical therapies, but more invasive options, including surgery, are required in severe refractory cases. Resective procedures—such as total colectomy with ileorectal anastomosis or segmental colectomy—offer limited long-term functional outcomes and are associated with significant morbidity [45, 46].

MACE offers an alternative by enabling self-administered antegrade colonic irrigation [9]. In its original form, the procedure involves resecting the appendix—while preserving its arterial supply—and creating a submucosal tunnel in the cecum, where the distal appendix is sutured. The proximal end is externalized at the skin level to form a stoma through which antegrade irrigation is performed using a catheter. When the appendix is unavailable, alternative approaches using the terminal ileum, cecum [13], or distal colon [47] have been described. More recently, minimally invasive laparoscopic or percutaneous endoscopic techniques have been developed with comparable results [13, 23]. Initially introduced for the treatment of FI in pediatric patients [9, 48, 49], MACE was later adopted for constipation as well. Pediatric studies have demonstrated long-term success rates of 78%–93% [18, 50], while evidence in the adult population remains limited and more heterogeneous. The reality is, however, that many adults with MACE have transitioned from the pediatric population, and therefore, the two groups are not always distinct. Nevertheless, our review focused exclusively on a pure adult population, thereby complementing the limited data currently available.

In adults, MACE is used to treat constipation, FI, or both. Although most published studies are small, single-center case series [50], our review indicates that a substantial proportion of adult patients (46.9%–92.9%) continued using irrigations at the end of follow-up, in line with a previous meta-analysis reporting success rates of approximately 74% (range, 66%–83%) at an average follow-up of 39 months [10, 51]. From a technical standpoint, appendicostomy should be preferred when the appendix is available, as it is associated with lower rates of stomal stenosis compared to ileal channels. Minimally invasive and percutaneous approaches may be considered in selected patients, but current data suggest a higher risk of pain or local complications.

Important considerations for the clinical implementation of MACE are its cost-effectiveness and adequate patient selection. While formal cost-effectiveness analyses in adults are limited, the procedure may reduce long-term healthcare utilization by decreasing the need for chronic medications, hospitalizations for complications of constipation or FI, and repeated interventions. Patient selection remains crucial. MACE appears to be most beneficial for adults with refractory constipation or FI who have not responded to conservative therapies, including dietary management, laxatives, and transanal irrigation (TAI), and who possess adequate manual dexterity and motivation to perform regular antegrade irrigations. Patients with severe neurogenic bowel dysfunction (e.g., spinal cord injury, spina bifida) may particularly benefit, while outcomes could be less predictable in those with progressive neurological decline where declining mobility is expected. Classic surgical options—such as segmental, subtotal, or total colectomy—achieve success rates of 65%–100% but are associated with substantial morbidity, including diarrhea (46%), abdominal pain (41%), de novo incontinence (21%), and small bowel obstruction (15%) [52–54]. Similarly, sacral nerve stimulation carries a procedural complication rate leading to reoperation in 13%–34% of patients and a device removal rate of 8%–23% at a mean follow-up of 31 months, most commonly due to lack of efficacy, infection, lead-related issues, or pain at the implant site and from stimulation [55]. In comparison, MACE represents a less invasive alternative with favorable long-term outcomes.

Indeed, MACE is most directly compared with TAI, another non-pharmacological technique aimed at improving bowel function. TAI shares similar indications and mechanisms of action with MACE and has demonstrated sustained efficacy in 60%–70% of patients, along with improved QOL in both the short and long term [56–58]. Compared to MACE, which showed a 75.1% long-term success rate in our pooled analysis, TAI offers comparable functional outcomes with the advantage of being less invasive and fully reversible. However, MACE and TAI should not be considered mutually exclusive, but rather sequential steps within a therapeutic algorithm tailored to the severity of symptoms, patient compliance, and response to previous treatments. TAI is best suited as a first-line option due to its non-invasive nature and ease of use, particularly in compliant patients with preserved dexterity (e.g., those with low anterior resection syndrome or mild neurogenic bowel dysfunction). MACE, conversely, represents a later-stage intervention for patients who fail or do not tolerate TAI, or for those with anatomical or severe neurological conditions that render TAI ineffective (i.e., selected cases of severe or refractory LARS). More recently, alternative MACE techniques involving the descending colon have been described. Notably, Macedo et al. introduced a method using a catheter placed in the left sigmoid colon to create a stoma for distal colonic irrigation, thereby avoiding the use of the appendix [47]. Although the indications may overlap, left-sided procedures differ significantly from classic MACE and were therefore excluded from our systematic review [59].

Failure of the MACE procedure—traditionally defined as the need for conversion to permanent colostomy—ranged from 0% to 29.0%, with stoma-related complications representing a major contributing factor [25]. Stomal stenosis, often requiring dilatation or surgical revision, occurred six times more frequently following appendicostomy compared to ileal appendicostomy. Local pain, particularly after percutaneous endoscopic approaches [13], also contributed to failure, potentially due to injury of the ilioinguinal or iliohypogastric nerves or colonic traction from endoscopic fixation. Other reported causes included persistent FI, stoma leakage, and progression of underlying neurological disorders that impeded effective irrigation [26]. It is important to note that while conversion to colostomy or cessation of antegrade irrigation provides an objectively measurable endpoint, this definition may oversimplify clinical reality and fail to fully capture patient-centered success. In some cases, patients may successfully transition to TAI after stoma closure, whereas in others—particularly those with severe motor disability—a permanent colostomy may represent the most appropriate and effective long-term solution. From the patients’ perspective, functional outcomes such as symptom relief and QOL improvement are arguably more relevant than technical continuation of irrigation or avoidance of colostomy. Therefore, definitions of success and failure should integrate both functional improvement and sustained use of MACE, rather than relying solely on procedural endpoints. Most studies reported improvements in bowel function and patient-reported QOL, supporting the view that the benefits of MACE extend beyond technical outcomes. However, interpretation is complicated by heterogeneity in assessment tools, including CCIS [35], KESS [34], and various QOL instruments, which limit direct comparisons.

This review is limited by the heterogeneity of the included studies (indications, techniques, follow-ups, retrospective and prospective designs) with relatively small sample sizes (3–75 patients per study, 404 patients in total). Only single-arm case series were available, precluding formal meta-analysis, publication bias assessment, or sensitivity analyses. Pooled results should therefore be interpreted as descriptive rather than inferential. Furthermore, functional outcomes were assessed using a wide range of scoring systems and quality-of-life instruments, which complicates direct comparison and pooling of results. In addition, although a formal risk of bias assessment was performed, its results were not homogeneous: most studies showed a moderate to high risk of selection and reporting bias, with only a minority judged as low risk. These limitations must be considered when interpreting the pooled findings, and observed success rates should be viewed as exploratory signals rather than definitive estimates. Future studies should aim to standardize outcome measures using validated scoring systems and QOL instruments to enable meaningful comparison. Comparative studies between MACE and TAI are needed to clarify their relative roles within treatment algorithms. In addition, long-term prospective registries could help identify predictors of success or failure, refine patient selection criteria, and explore cost-effectiveness. Finally, technical innovations—such as less invasive access techniques—should be investigated to reduce complications and improve the durability of treatment.

## Conclusions

MACE represents an effective and safe therapeutic option for the medium- and long-term management of severe constipation and FI in adult patients, particularly in those who have not responded to medical therapy and wish to avoid permanent colostomy. Over time, the majority of patients report satisfaction with the procedure, improved QOL, and maintain regular use of irrigations with satisfactory symptom control. However, no definitive conclusions can be drawn regarding the most effective MACE technique. While percutaneous endoscopic cecostomy offers the advantage of minimal invasiveness, it is frequently associated with postoperative pain and higher rates of conversion to colostomy. Conversely, surgical approaches involving the creation of a neo-appendix may provide better long-term outcomes, but they are associated with greater morbidity and a noteworthy risk of stomal stenosis.

**Table 5:** Long-term outcomes. *MACE*, Malone antegrade continence enema; *MACE success*, patients still performing irrigation out of the total patients available for the assessment at the end of follow-up. *CCIS*, Cleveland Clinic Incontinence Score; *BRQ*, Bowel-Related Questionnaire. *SF-36*, Medical Outcomes Study 36-Item Short Form Health Survey. *AMS*, American Medical Score; *CCCS*, Cleveland Clinic Constipation Score; *GIQLI*, Gastrointestinal Quality of Life Index; *KESS*, Knowles Eckersley Scott Symptoms Score; *FIQL*, Fecal Incontinence Quality of Life score; *QOL*, quality of life

Study	Follow-up (months)	MACE success, *n* (%)	Definite colostomy, *n* (%)	Functional scores/questionnaires used	Functional results
Hill et al. [12]	3–15	-	-	-	4 pts. resolved, 2 improved symptoms
Krogh et al. [21]	17 (1–39)	10/16 (62.5%)	0	Overall satisfaction, generic bowel function score (0–100)	75% reported satisfaction
Rongen et al. [31]	18 (12–23)	8/12 (66.6%)	2 (16.7%)	CCCS, State-Trait Anxiety Inventory, Zung Depression Scale, State-Trait Anxiety scale, Nottingham Health Profile	Improvement CCCS, depression and anxiety
Teichman et al. [33]	54 ± 6	5/6 (83.3%)	1/6 (16.7%)	Overall satisfaction	Generic improvement
Lees et al. [32]	36 (13–140)	15 (46.9%)	9 (29.0%)	-	-
Hirst et al. [22]	6 (3–51)	13/15 (86.7%)	0	CCIS, BRQ for ODS, SF-36	Improvement CCIS, BRQ. No improvement SF-36
Portier et al. [28]	27–35	26/28 (92.9%)	0	-	-
Lefèvre et al. [29]	26 (10–85)	18/22 (81.8%)	1/22 (4.5%)	Overall satisfaction, SF-36	Improved social function, role emotional and mental health 64% reported excellent satisfaction
Uno [23]	9 (1–18)	-	-	-	-
Altomare. et al. [27]	44 (33–66)	9/11 (81.8%)	1/11 (9.1%)	AMS, CCCS, GIQLI	All scores improved
Meurette et al. [30]	55 ± 36	13/25 (52.0%)	5/25 (20%)	KESS, GIQLI	KESS similar, GIQLI improved
Chéreau et al. [24]	48 (4–110)	64/70 (91.4%)	0	CCIS, GIQLI, SF-36	CCIS improved, GIQLI and SF-36 in normal ranges
Duchalais E. et al. [13]	12	11/18 (61.1%)	-	KESS, GIQLI	All scores improved
Sturkenboom R. et al. [14]	43 ± 26	22/30 (73.3%)	8/30 (26.7%)	Malone continence scale, CCCS, SF-36, Karnofsky, VAS, Vaizey survey	Malone: 61.1% full-partial success Vaizey: 60% partial success CCCS: 61.5% full-partial success SF-36: no difference with general pop
Ricard J et al. [20]	-	50/67 (74.6%)	5/67 (7.5%)	KESS, CCIS, GIQLI	All scores improved
Brinas P. et al. [25]	33 (0.7–126)	16/23 (69.6%)	5/23 (21.7%)	KESS, CCIS, FIQL	KESS and CCIS unchanged FIQL improved
Spinelli M. et al. [26]	14, 84, 121	3/3 (100%)	0/3 (0%)	-	-

## Data Availability

No datasets were generated or analysed during the current study.
